# Clinical efficacy evaluation of Erlotinib Combined with Concurrent Chemoradiotherapy in the treatment of locally advanced Pancreatic Cancer

**DOI:** 10.12669/pjms.38.1.4150

**Published:** 2022

**Authors:** Ci Liu, Haobin Yu, Yue-hong Hou, Zhen-lin Gao, Ya-jing Zhang

**Affiliations:** 1 Ci Liu, Department of Internal Medicine, Beijing Water Resources Hospital, Beijing, 100036, China; 2 Haobin Yu, Dept. of Cancer Nutrition & Metabolic Therapy, No.3 Ward of Oncology, Anhui Provincial Cancer Hospital, West Branch of the First Affiliated Hospital of USTC, Division of Life Sciences & Medicine, University of Science & Technology of China, Hefei 230001, Anhui P.R. China.; 3 Yue-hong Hou, Department of Oncology, Shijiazhuang First Hospital, Shijiazhuang, Hebei, China; 4 Zhen-lin Gao, Department of Oncology, Shijiazhuang First Hospital, Shijiazhuang, Hebei, China; 5 Ya-jing Zhang, Department of Oncology, Shijiazhuang First Hospital, Shijiazhuang, Hebei, China

**Keywords:** Concurrent chemoradiotherapy, Erlotinib, Locally advanced pancreatic cancer, Treatment

## Abstract

**Objective:**

To evaluate the clinical effects of erlotinib combined with concurrent chemoradiotherapy in the treatment of locally advanced pancreatic cancer.

**Methods:**

Eighty patients with locally advanced pancreatic cancer who attended Shijiazhuang People’s Hospital or Anhui Cancer Hospital between January 2018 and January 2020 were randomly divided into two groups, with 40 cases in each group. Patients in the control group were treated with concurrent chemoradiotherapy, while those in the experimental group were treated with erlotinib tablets based on the treatment regimen of the control group. Anti-tumor efficacy evaluation was conducted for all patients in both groups, and the adverse drug reactions, improvement of performance status after treatment were compared and analyzed between the two groups.

**Results:**

The overall response rate of the experimental group was 47.5%, which was significantly better than the 25% of the control group (p=0.03). The incidence of adverse drug reactions in the experimental group was 40%, while that in the control group was 30%. The incidence of adverse drug reactions in the experimental group was higher than that in the control group, but there was no statistical significance (p=0.34). Moreover, the improvement rate of performance status score in the experimental group was significantly higher than that in the control group (p=0.00).

**Conclusion:**

Erlotinib combined with concurrent chemoradiotherapy has been preliminarily proved to be safe and effective in the treatment of locally advanced pancreatic cancer, which can improve the physical condition of patients to a certain extent without significantly increasing adverse reactions.

## INTRODUCTION

Pancreatic cancer, as a common malignant tumor of the digestive system, has gradually surpassed breast cancer in its incidence, accounting for the third place in the total number of cancer deaths in the United States.^1^ Its high mortality can be attributed to its biological characteristics, difficulty in early diagnosis, and susceptibility to recurrence and metastasis.^2^ Patients with pancreatic cancer have a poor prognosis, with a 5-year survival rate of only 8%, and most of them will relapse even after surgical resection.^3^ Ideal results are difficult to obtain for patients with locally advanced pancreatic cancer by surgical resection.^4^ Radiotherapy is a commonly used method for locally advanced pancreatic cancer. However, pancreatic cancer has poor sensitivity to radiotherapy, and poor results may be obtained by radiotherapy alone.^5^ Patients with locally advanced pancreatic cancer can benefit from chemotherapy and optimal supportive care.^6^ For locally advanced pancreatic cancer, chemotherapy remains the standard of care. In addition, gemcitabine-based combination chemotherapy is the most effective method for the treatment of pancreatic cancer, but there is no unified standard for which drug to combine with.^7^ Erlotinib is a novel epidermal growth factor receptor (EGFR) tyrosine kinase inhibitor that can inhibit tumor growth, which is often used clinically to treat advanced EGFR-mutant lung adenocarcinoma.^8^ In this study, a prospective cohort study was used to evaluate the clinical efficacy of erlotinib combined with concurrent chemoradiotherapy for locally advanced pancreatic cancer. Detailed results are reported as follows.

## METHODS

A quasi experimental study was used in this study. Twenty cases of patients with locally advanced pancreatic cancer admitted to Shijiazhuang People’s Hospital and 60 cases of patients with locally advanced pancreatic cancer admitted to Anhui Cancer Hospital from January 2018 to January 2020 were selected according to the principle of random sampling. They were randomly divided into two groups according to the random number table method, with 40 cases in each group. There was no significant difference between the two groups in gender, age, tumor stage, tumor site and other general information, which was comparable (Table-I).

**Table I:** Comparative analysis of general data between the two groups ( ±S) n=40.

Indicators	Experimental group	Control group	t/χ^2^	P
Age	55.74±6.42	57.63±7.11	1.25	0.22
Male (%)	26 (65%)	23 (57.5%)	0.47	0.49
***TNM staging***				
T1-T3N2	23 (57.5%)	21 (52.5%)	0.20	0.65
T4NX	17 (42.5%)	19 (47.5%)	0.23	0.65
***Tumor location***				
Pancreatic head	14 (35%)	13 (32.5%)	0.06	0.81
Pancreatic body	21 (52.5%)	23 (57.5%)	0.20	0.65
Pancreatic tail	5 (12.5%)	4 (10%)	0.13	0.72

P>0.05.

### Ethical approval:

The study was approved by the Institutional Ethics Committee of Beijing Water Resources Hospital at February 20, 2019 (No.:152777127), and written informed consent was obtained from all participants.

### Inclusion criteria:

• Patients diagnosed with pancreatic cancer by CT, MRI and other imaging examinations and histopathological biopsies.^9^
• Patients in the locally advanced stage (T1-T3N2/T4NX).
• Patients with an estimated lifetime ≥6 months.
• Patients whose family members are willing and able to cooperate to complete the study and have good treatment compliance.
• Patients who have no contraindications to the drugs used in this study.
-Newly treated patients who are > 18 years old and have not received chemoradiotherapy.

### Exclusion criteria:

-Patients with pancreatic endocrine carcinoma of pathological type.
• Patients with mental or cognitive dysfunction and unable to cooperate with the implementation of this study.
• Patients with severe organic or congenital diseases of heart, liver and kidney that may be life-threatening.
• Patients complicated with autoimmune diseases and inflammatory diseases.
• Patients who have taken relevant drugs such as immunosuppressants and hormones that affect the study in the near future.
• Patients with unstable vital signs such as blood pressure, blood oxygen, heart rate and respiratory rate.

### Treatment methods:

Blood cell analysis, liver function and renal function examination were performed in both groups, and abnormal indicators were corrected accordingly. During the treatment period, nutritional assessment was conducted, nutritional support treatment was given to patients with malnutrition, and basic treatment methods such as antiemetic correction of electrolyte disturbance were given to those with corresponding symptoms.

Patients in both groups received the same standard of nursing intervention. The control group was given a concurrent chemoradiotherapy regimen: For radiotherapy regimen, an Elektai X electron linear accelerator was used, and radiotherapy areas were divided as follows: 1. GTV: Primary pancreatic mass tissue; 2. CTV: Area where GTV expands outward by 5-10mm; 3. PTV: Area where CTV expands outward by 5mm. The prescribed dose of PTV was 50-50.4Gy/28-30 times, with five times/week. For chemotherapy regimen, gemcitabine was injected intravenously at a dose of 1000 mg/m2 for 30 minutes, once a week, with a course of treatment for three weeks and two consecutive courses.^10^ Erlotinib was given in addition to that in experimental group: 100 mg/d, oral administration, qd, with a cycle of four weeks. Erlotinib tablets were taken continuously until the disease progresses or an intolerable adverse reaction occurred. The maximum follow-up time for patients in both groups was six months. And case data collection ceased in August 2020.

### Observation indicators:

***Efficacy evaluation:*** All patients underwent anti-tumor efficacy evaluation after the treatment, blood routine/liver and kidney function/electrolytes were reviewed weekly and nutritional assessments were performed. After treatment, the tumor was evaluated according to the Response Evaluation Criteria in Solid Tumors 1.0 (RECIST1.0)^11^: Complete remission (CR): the lesion disappeared completely; Partial remission (PR): the total measured diameter of the target lesion decreased by 30% from the baseline; Stable disease (SD): the total change of the greatest diameter of target lesions was between PR and SD. Progression disease (PD): the sum of the long diameters of all target lesions increased by at least 20%, and the absolute value of the increase in the sum of long diameters increased by more than 5mm (or new lesions appeared). Overall response rate = (CR + PR) cases/total cases × 100%.

***Evaluation of adverse drug reactions:*** adverse drug reactions occurring in the two groups within one month after administration were recorded, including erythra, leukopenia, erythropenia, thrombocytopenia, gastrointestinal symptoms and other adverse reactions.

***Performance status score:*** ECOG score^12^ was used to observe the improvement of performance status before and after treatment: improvement (≥ 1 point reduction), stability (score unchanged), deterioration (≥ 1 point increase).

### Statistical analysis:

All the data were statistically analyzed by SPSS 20.0 software, and the measurement data were expressed as (x̄± s). Two independent sample t-test was used for inter-group data analysis, paired t-test was used for intra-group data analysis, and χ^2^ test was adopted for rate comparison. P<0.05 indicates a statistically significant difference.

## RESULTS

The comparative analysis of the therapeutic effect of the two groups is shown in Table-II, indicating that the overall response rate of the experimental group was 47.5% after treatment, which was significantly better than the 25% of the control group, with a statistically significant difference (p=0.03).

**Table II:** Comparative analysis of therapeutic effect between the two groups (X̅ ±S) n=40.

Group	CR	PR	SD	PD	Overall response rate
Experimental group	2	17	17	4	19 (47.5%)
Control group	0	10	23	7	10 (25%)
χ^2^					4.38
P					0.03

P<0.05.

Incidence of adverse drug reactions in the two groups after treatment was compared and analyzed, indicating that the incidence rate of adverse reactions in the experimental group was 40%, and that in the control group was 30%. The incidence rate of adverse drug reactions in the experimental group was higher than that in the control group, but there was no statistical significance (p=0.34) (Table-III). After treatment, the improvement rate of the performance status score of the experimental group was significantly higher than that of the control group (p=0.00) (Table-IV).

**Table III:** Comparative analysis of adverse drug reactions of the two groups after treatment ( ±S) n=40.

Group	Erythra	WBC reduction	RBC reduction	PLT reduction	Gastrointestinal reaction	Incidence rate
Experimental group	3	5	1	3	4	16 (40%)
Control group	0	4	2	3	3	12 (30%)
χ^2^						0.88
P						0.34

p<0.05

**Table IV:** Comparative analysis of performance status scores (ECOG) of the two groups before and after treatment (X̅ ±S) n=40.

Group	Improvement*	Stable	Deterioration
Experimental group	25	11	4
Control group	12	19	9
χ^2^	8.50	3.41	2.30
P	0.00	0.06	0.13

* p<0.05.

## DISCUSSION

The incidence rate of pancreatic cancer has been increasing year by year in recent years. However, many patients have been diagnosed with advanced disease by the time it is discovered.^13^ Currently, surgical resection, radiotherapy and chemotherapy are taken as the main clinical treatments for pancreatic cancer.^14^ For the treatment of locally advanced pancreatic cancer, radiotherapy or chemotherapy is the most commonly used treatment in the clinic. Combined targeted therapy can improve the efficacy of patients with pancreatic cancer due to the multifactorial nature of pancreatic cancer.^15^ For the chemotherapy of pancreatic cancer, gemcitabine monotherapy for pancreatic cancer has been the standard treatment for many years.^16^ According to Alexander et al.^17^, chemotherapy combined with radiotherapy is a safe and well-tolerated treatment regimen for locally advanced pancreatic cancer, which can improve efficacy.

Erlotinib exerts its unique therapeutic effect in blocking downstream signal transduction, reducing the activity of tyrosine kinase and the adhesion of tumor cells, and promoting tumor cell apoptosis by binding to epidermal growth factor receptors.^18^ Epidermal factor receptors may lead to tumor cell invasion, proliferation and metastasis. The study of new effective treatment strategies for tumors and the molecular mechanisms driving tumorigenesis of pancreatic cancer may provide new treatment opportunities for patients with pancreatic cancer and assist them in choosing the optimum treatment method.^19^ In advanced pancreatic cancer, there is an overexpression of epidermal factor receptor that affects the progression of the disease.

In this study, erlotinib combined with concurrent chemoradiotherapy is confirmed to have an overall response rate of 47.5%, while that of concurrent radiotherapy alone is 25%, suggesting that erlotinib combined with concurrent chemoradiotherapy has more obvious advantages for locally advanced pancreatic cancer (p=0.04). Studies have shown that erlotinib can increase the expression of gemitabine-induced NF - κ B and its binding activity to tumor cell DNA. Gemcitabine combined with erlotinib has a synergistic anti-proliferation effect on pancreatic cancer cell lines.^20^ In addition, it was pointed out by McGuigan et al. that erlotinib combined with gemcitabine is the first-line treatment for locally advanced pancreatic cancer.^21^ Lee et al.^22^ believed that erlotinib could enhance the sensitivity of gemcitabine pancreatic cancer cells. Our study also found that, after erlotinib treatment, the adverse reactions in the experimental group are slightly higher than those in the control group (40%, 30%), but there is no significant difference (p=0.34). Previous studies have shown that, compared with erlotinib alone, the adverse reactions of erlotinib combined with radiotherapy or chemotherapy have no significant increase in grade 3-4 toxicity except nausea.^23^ The improvement of the quality-of-life score in the experimental group is significantly higher than that in the control group (p=0.00). The study conducted by McCleary et al.^24^ concluded that erlotinib combined with radiotherapy or chemotherapy had no significant effect on the overall prognosis of patients with metastatic pancreatic cancer, but the quality of life was significantly improved.

### Limitations of this study:

Nevertheless, deficiencies still exist in this study: only the overall category of patients with locally advanced pancreatic cancer were studied due to the small sample size and short follow-up time, and no detailed comparative analysis has been conducted on different clinical stages of patients.

### Recommendation:

Further prospective studies are being carried out to reconfirm the therapeutic effect and long-term outcome of erlotinib combined with concurrent chemoradiotherapy in locally advanced pancreatic cancer by continuously enriching data, increasing follow-up time, and conducting further analysis on patients with different clinical stages.

## CONCLUSION

Erlotinib combined with concurrent chemoradiotherapy has been preliminarily proved to be safe and effective in the treatment of locally advanced pancreatic cancer, which can improve the physical condition of patients to a certain extent without significantly increasing adverse reactions.

### Authors’ Contributions:

**CL and HY** designed this study, prepared this manuscript, and are responsible and accountable for the accuracy or integrity of the work.

**YH and ZG** collected and analyzed clinical data.

**YZ** significantly revised this manuscript.
